# Teamwork in primary care: perspectives of general practitioners and community nurses in Lithuania

**DOI:** 10.1186/1471-2296-14-118

**Published:** 2013-08-15

**Authors:** Lina Jaruseviciene, Ida Liseckiene, Leonas Valius, Ausrine Kontrimiene, Gediminas Jarusevicius, Luís Velez Lapão

**Affiliations:** 1 Department of Family Medicine, Lithuanian University of Health Sciences (LUHS), Mickeviciaus 9, Kaunas LT 44307, Lithuania; 2 Department of Cardiology, Lithuanian University of Health Sciences, Mickeviciaus 9, Kaunas LT 44307, Lithuania; 3 WHO Collaborating Center for Health Workforce Policy and Planning, Instituto de Higiene e Medicina Tropical, Universidade Nova de Lisboa, Portugal, Rua da Junqueira 100, Lisbon 1349-008, Portugal

**Keywords:** Teamwork, General practitioners, Community nurses, Primary health care, Lithuania

## Abstract

**Background:**

A team approach in primary care has proven benefits in achieving better outcomes, reducing health care costs, satisfying patient needs, ensuring continuity of care, increasing job satisfaction among health providers and using human health care resources more efficiently. However, some research indicates constraints in collaboration within primary health care (PHC) teams in Lithuania. The aim of this study was to gain a better understanding of the phenomenon of teamwork in Lithuania by exploring the experiences of teamwork by general practitioners (GPs) and community nurses (CNs) involved in PHC.

**Methods:**

Six focus groups were formed with 29 GPs and 27 CNs from the Kaunas Region of Lithuania. Discussions were recorded and transcribed verbatim. A thematic analysis of these data was then performed.

**Results:**

The analysis of focus group data identified six thematic categories related to teamwork in PHC: the structure of a PHC team, synergy among PHC team members, descriptions of roles and responsibilities of team members, competencies of PHC team members, communications between PHC team members and the organisational background for teamwork. These findings provide the basis for a discussion of a thematic model of teamwork that embraces formal, individual and organisational factors.

**Conclusions:**

The need for effective teamwork in PHC is an issue receiving broad consensus; however, the process of teambuilding is often taken for granted in the PHC sector in Lithuania. This study suggests that both formal and individual behavioural factors should be targeted when aiming to strengthen PHC teams. Furthermore, this study underscores the need to provide explicit formal descriptions of the roles and responsibilities of PHC team members in Lithuania, which would include establishing clear professional boundaries. The training of team members is an essential component of the teambuilding process, but not sufficient by itself.

## Background

The 1978 Alma-Ata declaration emphasising a team approach in primary health care (PHC) marked the beginning of a new era [1]. Nowadays PHC is inconceivable as anything other than a competently functioning health care team [2,3]. A team approach in PHC has proven advantageous in achieving better outcomes [4-6]. Such outcomes include reducing health care costs due to a lower number of hospitalisations [7], satisfying patients’ needs [8,9], ensuring continuity of care [10,11], increasing job satisfaction for health providers [12] and using human health care resources more efficiently [2,9].

Research indicates that the initial step in implementing a team-based approach in PHC involves a shift from physician-centred practices to “goal-oriented” practices defined by a physician-led team [2]. The PHC team then evolves as its structure progressively integrates different health professionals [13] to work with the physician, then professionals in health-related fields [10] and finally various laypersons [14]. Studies demonstrate that health care processes and outcomes improve when non-physician team members provide important components of care [2,15-17].

Boon et al. [18] propose a conceptual framework that identifies seven types of team oriented health care practice and situates them on a continuum ranging from least-integrated to most-integrated. According to the framework, the continuum of team-oriented health care practice is represented by parallel, consultative, collaborative, coordinated, multidisciplinary, interdisciplinary and integrative models. Increasing integration across the continuum is reflected by changes in structure, process and outcomes, as well as by the team’s health care philosophy. A well-functioning team at one extreme on this continuum (low integration) could possess a different set of key characteristics than a well-functioning team at the other extreme (high integration) [18,19]. Research indicates that promoting team-oriented policies is the most effective management behaviour in organisations, where teams constitute the main work structure [20]. However, little is known about how these policies should differ depending on the existing level of team integration.

The Alma-Ata conference, which took place in the former Soviet Union, established the cornerstones of the modern understanding of PHC. Paradoxically, however, post-Soviet countries struggled more than Western countries did in implementing these principles in practice. The idea of collaboration within a PHC team as well as between teams did not correspond to the perspective intrinsic to the Semashko model predominating in these countries, which was characterized by centralisation and fragmentation of care. The Semashko model’s aspirations to provide universal health coverage free of charge resulted in financially burdened health systems due to weak PHC and an emphasis on in-patient care with high demand for specialists, little continuity and poor coordination of care [21]. Inter-professional collaboration eventually emerged as an issue, primarily during the health care reforms that followed the collapse of the Soviet Union [22-25].

One of the main focuses of PHC reform in Lithuania was strengthening inter-professional collaboration [26]. In reality, however, aspiration was scaled back to merely introducing the institution of general practice and recognising it as a medical career, followed by (re)training general practitioners (GPs). District nurses changed their titles to community nurses (CN) by law, but the content of their work changed only superficially – they mainly continued acting as physicians’ assistants. Some post-Soviet countries, i.e., Estonia, shifted a considerable part of GP responsibilities to CNs [27]. In contrast, Lithuanian CNs continued to work in a traditional hierarchical relationship with GPs. Furthermore, Lithuanian CNs typically work in the same offices with GPs and do not provide independent patient consultations [28].

Actually there are several types of health care providers who formally are recognized as primary health care professionals: GPs, CNs, psychiatrists, mental care nurses, and social workers [29]. GPs and CNs form PHC teams and work together in PHC centres, while psychiatrists, mental care nurses and social workers form mental health care teams working in independent mental health centres.

Although the concept of the PHC team is widely acknowledged in Lithuanian health policy, a formal framework for such team practice is lacking and wide organizational diversity is observed in PHC institutions. The PHC team usually includes GPs, CNs and often clinical administrative employees but seldom social workers. Practicing GPs and/or CNs are usually in charge of the management of the PHC setting.

A recent study that examined mental health service provision in Lithuanian PHC found an extremely low level of CN involvement. When treating mentally ill patients, CNs rarely or never helped 72.8% of GPs in assessing the mental status of patients and rarely or never assisted 62.2% of GPs in delivering home care services [30]. Research indicates that Lithuanian CNs perceive laboratory test-taking, clinical administration and sorting patients for physician consultation as the essential domains of their professional activity. Nonetheless, for example, CNs evaluate provision of psychosocial services in the community as an additional duty [31].

These findings suggest that a biomedical approach prevails in Lithuanian PHC and that PHC teams do not work collaboratively. This would situate them on the low-integration side of the spectrum identified by Boon et al. [18,19]. The need to improve teamwork for PHC provision in Lithuania is recognized [30,32] and some efforts have been made to make teamwork more effective [33]. However, a survey of the available evidence indicates that there apparently have been no previous studies assessing the experiences of PHC providers on this issue, nor have there been any attempts to identify the key components of team-oriented health care practice promotion in the Lithuanian PHC context with low team integration. Therefore this study aimed to explore the practice of teamwork as viewed by GPs and CNs for a better understanding of the phenomenon of teamwork in Lithuanian PHC and for the purpose of identifying opportunities to strengthen team-oriented health care practice in the context of low team integration.

## Methods

The research presented in this paper reflects the qualitative component of a larger project titled *Intersectorial collaboration solving health care problems in social risk families*. The two-year (2012–2013) project, financed by the Lithuanian Agency of Sciences, aims to assess the potential of collaborative working between PHC and social services in Lithuania to respond better to health and social care needs of families at social risk.

The project involves three research components: focus groups with GPs and CNs to explore experiences with teamwork in the PHC context; a cross-sectional survey of PHC professionals and social sector professionals to identify current collaborative practices as well as factors associated with more effective collaboration; and vignettes of PHC providers and representatives of the social sector to examine the quality of their performance in addressing the care needs of social risk families and to identify existing collaborative patterns the between health care and social sectors.

The scope of this paper which aims to identify the constituents of teamwork in the transforming Lithuanian PHC is confined to data obtained from focus groups conducted with GPs and CNs in the Kaunas region. This region, Lithuania’s most central, is highly urbanised, with less than one fifth of residents living in rural areas. The population of the Kaunas region constitutes almost 15% of the total population of Lithuania. Economic indicators in the region (e.g. salary) are equal to the average in Lithuania.

There were 49 primary health care centres in the Kaunas region providing primary health care services under contract with the National Health Insurance Fund in the fall of 2011. Public and private institutions working under contract with the National Health Insurance Fund provide free PHC services to all insured patients. Certain populations such as children under the age of 18, students, and unemployed people are State-insured. The financing for all PHC facilities consist of capitation fees (approximately 70%) and production incentive payments (approximately 30%).

Invitations to take part in this study were sent to 25 PHC institutions in Kaunas region. They were selected with the aim of capturing the perspectives of primary health care providers working at large, medium and small facilities as well as in public and private PHC settings. Eleven PHC institutions agreed to take part in this survey and to distribute the information about the focus group discussions among employees. There were three large, three medium, and five small institutions, and four public and seven private institutions. Based on legal description of PHC team professionals, only GPs and CNs were invited to take part in this study [29].

As all GPs and CNs with distinct teamwork experience were voluntarily involved in the study and presumably had strong interest in the issue, we gave a priority to focus groups discussions instead of individual interviews. It was believed that group interactions could be richer and deeper than those occurring in individual interviews, [34] helping to elicit a diverse range of opinions and experiences [35].

The Bioethics Committee of the Lithuanian University of Health Sciences approved this qualitative study in 2012.

### Participants

A total of 56 PHC professionals [29 GPs and 27 CNs] participated in this study. Table 1 presents participant information. The focus group sessions were scheduled separately for GPs and CNs. It was believed that greater homogeneity in the backgrounds and status of health providers within the hierarchy of health settings would have a favourable effect on fostering open communication among study participants.

**Table 1:** Demographic breakdown of focus group participants

**Indicator**	**Number**
**Gender**	
Male	4
Female	52
**Age**	
Younger than 50	32
50 and older	24
**Profession**	
General practitioner (GP)	29
Community nurse (CN)	27
**Location of practice**	
Urban	45
Rural	11
**Type of practice**	
Public	36
Private	20
**Size of primary health care institution**	
Large (20,000 or more patients listed)	22
Medium (5,000 or more but less than 20,000 patients listed)	21
Small (less than 5,000 patients listed)	13
**Total number**	56

### Data collection

Two facilitators moderated each discussion. Both were female GPs. The first facilitator (the principal investigator) has a degree in applied sociology as well as being a medical doctor; the second facilitator has completed introductory training in qualitative research methodology and data analysis. As both facilitators have their own practices in the same geographical area, they were acquainted with some focus group participants, but there were no participants with whom they had personal relationships.

Study participants provided written informed consent. Focus group discussions lasted for about 1.5 hours to 2 hours. They were audio-taped with the participants’ permission. All participants were guaranteed confidentiality, and were told how data collected during the study would be used. Six discussions were conducted in total – three with GPs and three with CNs.

The focus group discussions followed a semi-structured topic guide (see Topic guide for focus group discussions). The guide included open-ended questions prompting participants to describe their perceptions of the PHC team in general, their experiences of everyday collaboration among GPs and CNs, their perceptions of factors negatively and positively affecting such collaboration, and their thoughts about their personal roles in an effective PHC team.

Topic guide for focus group discussions

1. How would you describe a PHC team?

2. How do the GPs and CNs collaborate in practice? Could you tell us from your experience, how do you engage in teamwork on a daily basis?

3. What negatively affects collaboration between GPs and CNs?

4. What favourably affects collaboration between GPs and CNs?

5. How do you see your role in an effective PHC team?

6. Any other comments?

The moderator encouraged experiential narratives. After each discussion, the two facilitators considered whether the topic guide should be refined in light of points made by the focus group participants. The question, “How do you see your role in an effective PHC team?” later was complemented with the prompt, “Could you describe your role in the ideal PHC team?” Although the core areas of discussion remained the same throughout the study, the format of each focus group discussion differed as along with the main aspects indicated in topic guide each focus group discussion raised different insights. When the discussions concerned different organizational practice in the home organizations of participants, such as payment models for team members, the interactions in the groups were especially vivid.

### Analysis

Each focus group discussion was transcribed verbatim. The thematic analysis [36] was initiated after all six focus groups were completed. The codes from the transcripts were generated in a systematic manner for the entire dataset by reviewing the data line by line. The terms selected for coding were as similar as possible to the participants’ own choice of words. Coding was performed by two independent researchers, and after initial coding was completed, the two coded transcripts were systematically compared. More than 80% of codes in both transcripts were similar; the remaining divergences were discussed by the research team until consensus about coding was reached. Closely related codes were arranged in thematic categories, and the final themes were formulated based on these groupings. They were then reviewed, refined, named and illustrated with quotations from the discussions. The data analysis was based on the inductive approach.

A bracketed ellipsis, or […], is used in the quotations presented below to indicate the omission of words, and an unbracketed ellipsis, or …, is used to indicate a reflective pause. Any interpretation by the researchers appears in brackets, e.g., [PHC team], indicating an effort to clarify the participants’ meanings. Focus group labels are provided to indicate the sources of quotations, e.g., “FG2GP” denotes the second focus group with GPs.

This paper analyses the study participants’ experiences related to teamwork. There were factors identified during the analysis that could be related to other topics, i.e., the transformation of CNs’ identity (confusion in roles, conflicting expectations, striving to explicitly delineate the scope of CNs’ work and the new professional boundaries). However, as this study was focused on aiming to identify aspects of teamwork in the Lithuanian PHC context, all other topics brought to light by the study were included in the analysis as contextual elements of teamwork in PHC rather than as separated themes.

The personal experiences of focus group participants differed in respect to the issues that were identified. As it might be related to different levels of their involvement in team-oriented health care practice as well as to different levels of team integration, we focused on the data that related to the question rather than on health provider satisfaction with these issues. Discrepancies between GPs’ perspectives and CNs’ perspectives in regard to different issues are nonetheless acknowledged in the text.

## Results

Study participants’ views about PHC teams were categorized in terms of the issues being addressed, and the categories were grouped under six major themes (Table 2). Each theme is presented below.

**Table 2:** Themes and categories emerging from a thematic analysis of a primary health care (PHC) teamwork

***Themes***	***Categories***	***Verbatim***
**Structure of the PHC team**	Main members of a PHC team	*“The team probably consists of a nurse and family physician, which is the most common primary care team model.” (FG1CN)*
	Optional members of a PHC team	*“Then I would count the receptionists…” (FG1GP)*
	Hierarchy in PHC teams	*“Our duties are different, but we should all be on a line, more horizontal.” (FG3GP)*
	Leadership in a team	*“Somebody should manage this team.” (FG3CN)*
**Synergy of PHC team members**	“Intangible” team unity	*“…nurse and physician […] – everything is operating automatically between them.” (FG1CN)*
	Common goals of team members	*“The goal is common – that patient should be stroked around from all sides; he should be happy…” (FG2GP)*
	Individual motivation to work in a team	*“This depends on the person. One would do everything, another would say: ‘I was told to do that and I will not do anything more.’ There are such people in a team too…” (FG3CN)*
	Trust between teammates	*“A physician should trust the nurse. If there is no trust, there is not any team.” (FG1CN)*
	Respect between teammates	*“Good relationships are most important, when you are working together and do not humiliate each other.” (FG1CN)*
	Executing commands of physician	*“And afterwards, in the office, she [the nurse] is doing what I am telling her to do.” (FG1GP)*
**Roles and responsibilities of team members**	Well-described, known roles of team members	*“Everybody should know what he must to do…” (FG2GP)*
	Confusion of roles	*“I do not know what her [CN] functions are. In fact we do not know what her duties are.” (FG1GP)*
	Overlap of responsibilities	*“If you take physician and nurse job descriptions, you see that many roles between them are overlapping.” (FG2GP)*
	Explicit boundaries of the roles and responsibilities of team members	*“The most important is not to intervene in treatment, since treatment belongs to the physician […]; the most important is to work within our own framework.” (FG2CN)*
	Delegation of tasks	*“Physician should say […] what he wants from the nurse.” (FG1CN)*
	Assumed individual responsibility	*“The nurse should know the boundaries of her work and take responsibility for their own actions.” (FG3GP)*
	Overlapping activities	*“It might they [nurses] are performing some tasks that belongs to us, but they are not fulfilling their own tasks.” (FG2GP)*
	Autonomous performance of one’s own duties	*“You should not say to a nurse, ‘You should do this or this.’ She should do this herself on the spot.” (FG1GP)*
	Sanctions for CN’s for overstepping boundaries	*“And during the meeting it was said that [name] is commenting on exam results. […] They made mud out of me so badly! […]” (FG1CN)*
	Positive expectations towards CN’s “doctoring”	*“She (CN) has my small stamp. I gave it to her that she could prescribe tests when I am absent” (FG2GP)*
	Differentiation of activities	*“For example, all certifications in our institution are written by the informational office.” (FG2GP)*
**Competency of primary care team members**	Appropriate knowledge and skills	*“Sometimes I hear […] our nurses who are consulting patients by phone […]. Once in a while […] such a consultation makes me cover my ears and not listen anymore. Really, the knowledge is outdated or inaccurate…” (FG3CN)*
	Supervision of competence	*“She [the CN] simply sometimes does not know […]. The administration should somehow control these things.” (FG2GP)*
	Necessity of training	*“I only would like to say that training is very much needed for nurses…” (FG2CN)*
	Training quality	*“We are going to the training as we would like to improve our competence, but we should officially question what we are receiving during this training.” (FG3CN)*
**Communications between PHC team members**	Communication as a tool to transmit work-related information	*“Since we [GPs and CNs] are working in separate offices, communication is very important […]. We are referring all the information to the physician […]. It‘s time saving for the physician and his consultation.” (FG2CN)*
	Communication as friendliness in the working environment	*“I worked in a private [health centre], but I didn’t like working there […]. There wasn’t enough communication…” (FG2CN)*
	Means to optimise communications in a team	*“We even have such a local telephone connection [between GP and CN offices]. If there is some question, we press a button and get in contact.” (FG2CN)*
	Inner language of team members	*“We understand one another in a glance.” (FG1GP)*
	Difficulties in mutual communication	*“They [CNs] react very sensitively […], even when you are talking very amiably […]. You see that she feels offended… somehow offended.” (FG2GP)*
	Communication strategies	*“I have heard about, when a physician who was close to the patient snapped out to the nurse everything he is thinking and… and had done this so awfully.” (FG1CN)*
**Organisational background for teamwork**	Synchronisation of compensation policies for team members	*“Our nurses have a fixed salary, so, the less patients I have, the happier she is, since she can sit and look through a window.” (FG2GP)*
	Workplace of team members	*“We do not have where to let them [CNs] sit down normally so we cannot ask them to perform tasks independently. It’s really so – there is no place to sit down.” (FG1GP)*
	Appropriate time for handling procedures	*“There should be some regulation on how many patients a physician can consult each day. Not how much he is consulting, but how much he should consult not losing quality.” (FG2GP)*
	Regulation of patient flows	*“When, instead of 12 patients with an appointment, 30 or even more come, this simply puts out of kilter all work in a team.” (FG2GP)*
	Work in stable districts	*“You feel pleased when you are going to your own district; you know all the tasks […]. It’s very important this many years to work in one place.” (FG3CN)*
	Introduction of innovations	*“Everything is on an electronic record system – if you had it, you wouldn’t need to rewrite information in referrals, recipes, etc…” (FG3GP)*
	Response to the needs of personnel	*- “We do not have an official time for lunch…” - “We have 15 minutes but, during this time, we take patients without appointments.” (FG2GP)*
	Training of personnel	*“We have training in our institution, lectures on how to communicate with patients […]. This is needed very much.” (FG2GP)*
	Team building initiatives	*“In the beginning [of institution’s activity] we had a lot of training on team work… at that time this seemed stupid and incomprehensive, but now it seems that it was worthy, this turned our heads in other direction. I mean, some understanding emerge…” (FG2GP)*
	Involvement of all personnel in non-clinical activity	*“All of us – doctors and nurses – were involved in preparation of office rules […] We had to go through the legal acts and prepare internal policies. We were not happy about that, but after that we knew each other better.” (FG3CN)*
	Best practice exchanges with other institutions	*“The heads of health care institutions should sit together and decide […] how to improve things […]. Now they are only competing with each other.” (FG2CN)*

### Structure of a PHC team

GPs and CNs were named as the main structural elements of a PHC team. Administrative employees and social workers were also mentioned as potential team members. Study participants described efforts by some PHC institutions to expand PHC teams by including new members such as social workers or secretaries. CNs said that GPs traditionally have a higher formal status on a team than nurses do; however, both GPs and CNs stressed that a team approach necessitates non-hierarchical relationships between teammates.

Team leadership emerged as an explicit issue only in discussions with CNs, who emphasised the need for better coordination of PHC team activities. They indicated that PHC teams do not actually have an explicit position of team leader. As per an unwritten rule, GPs are assumed to be the leaders of PHC teams, but CNs believe this is simply due to the traditionally higher status of GPs.

“We recognise the physician as a chief. But there is a big question, whether he should be a chief… Probably we simply inherited such an understanding from those [Soviet] years” (FG3CN).

### Synergy among PHC team members

Both GPs and CNs addressed the issue of synergy among team members. According to study participants, this intangible element in teamwork either can unify colleagues and lead them to function as a team or else it can emerge as a team gains experience over time. This element was described as a “*fist*” (FG3GP), a “*family*” (FG3CN), “*automatism*” (FG1CN), “*synchronisation*” (FG1GP) and “*a common attitude*” (FG2GP), and was perceived as a sign of an effective team culture.

Elements of synergy identified by study participants were trust and respect among team members and an individual commitment to work as part of a team. Some GPs emphasised the necessity for CNs to obey GPs in accomplishing the tasks delegated to them – this was perceived as a prerequisite for team synergy.

### Explicit descriptions of team members’ roles and responsibilities

In both GP and CN discussions, the most attention was paid to the functions of team members.

Study participants perceived that a primary step required for a team to work more effectively involved drawing clear distinctions between the roles and responsibilities of GPs and CNs, as well as defining what their contributions to patient care should be.

“They [the community nurses] do something, but the responsibility is only on the physician. She [a nurse] should have also her own responsibility. And when [I] did not handle what is her responsibility, it’s very helpful. Then she understands her own job and the tasks are distributed” (FG3GP).

In contrast, overlapping responsibilities, the lack of a formal distinction between roles, and a lack of well-described organizational procedures for different activities (especially when GPs and CNs work in one office) were reported to cause tension and confusion in a team. The expectations of one team member can differ from those of another, which causes conflicting expectations to flourish. Meanwhile, CNs expect to get their assignments from the physicians, they often feel “*serving doctors*” (FG2CN). As one CN stated, “*Physicians should say what they want from the nurses*” (FG1CN). On the other hand, physicians look forward to more independent behaviour on the part of the CNs and expect more active performance of duties by CNs.

Moreover, a diffusion of roles and responsibilities erodes boundaries between areas of professional expertise. GPs tend to protect the boundaries of their expertise; thus they usually tend to be intolerant of CNs who “intrude” in their field and fiercely criticise them: *“When she [CN] comes to me I can always see if something is wrong. […] Then I ask her: “So, you have ‘doctored’ again?” And then she begins to tell me what she has done…”* (FG2GP). Such intrusions may be independent prescriptions of tests, the provision of information to patients about test results, or suggestions about medications, to give some examples. Still, the attitude of GPs towards *“doctoring”* by CNs is not always consistent. GPs seem to welcome “*doctoring*” by CNs in specific situations (e.g., the absence of a physician, an overload of patients).

There were several examples of responsibilities being shifted in PHC teams, or other cases involving a differentiation of PHC activities. The current trend is to include new members into PHC teams (i.e., secretaries) as well as to introduce new services at PHC institutions (i.e., home care, palliative care, informational offices), which replace activities traditionally seen as GP and CN functions.

### Competency of PHC team members

Both the GP and CN discussions addressed the relevance of the competency of PHC members. “*Professionalism*” (FG3GP) on the part of PHC team members was identified as one of the essential features of teamwork.

However, both GPs and CNs reported that CNs often lack competence. Study participants suggested the necessity of assessing the competence of team members in an organisation and emphasised the importance of training. CNs underlined their need for continuing medical education programs that are of a higher quality and better suited to their needs.

### Communication between PHC team members

All study participants, especially CNs, emphasised that an important element of teamwork among PHC team members was communication, both professional and relational.

Study participants said that in their experiences, team communication functions in at least two ways. First, professional communication (“what to say”, in terms of sharing medical facts efficiently) is a tool to transmit information about patients and about the activities that have been performed. Second, relational communication between team members (“how to say it”, in terms of finding appropriate strategies for addressing issues that team members may have strong feelings about) seems to affect the working atmosphere favourably and increases job satisfaction.

According to study participants, team members are looking for means to facilitate the process of transmitting professional information. For one, they can use mutually known short symbols. As one CN stated, *“We understand what is written on this small piece of paper”* (FG3CN). Another means is simply the use of internal phone connections.

However, there seem to be rather frequent problems regarding relational communications between GPs and CNs. Both GPs and CNs face difficulties in finding effective strategies to communicate their expectations, delegate tasks and/or discuss certain negative experiences.

According to CN perceptions of the current situation, there has been a shift in relational communications between GPs and CNs. It seems to them that younger GPs tend to create an atmosphere for communicating in a team that has less hierarchy and involves more collaboration than older GPs do.

“*The younger physicians […], they are different […]. It might be more conscious […], friendlier […]. They are addressing us differently, communicating differently, … but I am not saying that all Soviet physicians are like this…” (FG2CN)*

### Organisational background for teamwork

Study participants indicated several issues concerning organisation-level factors influencing teamwork: financial motivation, optimal working conditions, better work organization and team-building initiatives.

Systems for calculating individual salaries differ at each institution. Apparently the desynchronisation of payment policies for different primary care team members could negatively affect the synergy in a team. For example, in some PHC institutions, the salary of a GP depends on the number of his/her listed patients (capitation), but the CN receives a fixed salary. Thus GPs tend to have more patients to service on their list, whereas CNs have no interest in this. Their salary does not increase, but the situation prompts them to work more intensely.

Furthermore, study participants perceived working conditions as important for teamwork. According to them, the managers of PHC institutions should revise internal office policies to define and eliminate futile segments of work, regulate the flow of patients more rigorously to assure sufficient time for handling different health care procedures, promote and introduce innovations (e.g., proper utilization of electronic health records) and, finally, remember the individual needs of PHC team members (e.g., assurance of short breaks during the working day).

Study participants also viewed the managers of health institutions as the primary initiators of team-building initiatives and as the people who should encourage widely shared best practices. Possibilities for strengthening the alliance among team members were not addressed explicitly during the focus groups discussions. However, the experiences of study participants revealed that continuous medical education sessions for all PHC team members at their own institutions and participation in making internal office policies could be instrumental in strengthening the sense of belonging to a team.

## Discussion

The results emerging from this study provide insights into the dynamics of PHC teamwork from the perspective of the PHC team members themselves. Six key themes were identified describing the framework for inter-professional teamwork in PHC. These themes concern the PHC team structure, synergy among PHC team members, explicit descriptions of the roles and responsibilities of team members, their competence to perform their designated duties, communications among team members and the organisational background for teamwork.

The resulting thematic model is based on these findings. It embraces formal, individual and organisational factors that might be suggested. Factors such as the explicit structure of a team, well-described functions and responsibilities of team members, and adequate competence of team members to perform designated duties constitute the formal framework, which could be referred to as the “hardware” for teamwork. The individual aspects, such as non-hierarchical relationships, respect and communications among allied teammates provide the behavioural “software” for teamwork. The formal and individual factors are interrelated; the organisational environment could have an enabling or inhibiting effect on the realisation of these factors.

Our suggested model reflects insights provided by Pullon et al. [37]. Based on findings from their qualitative study with New Zealand primary care physicians and nurses, the authors argue that intrinsic teamwork factors such as interprofessional respect, trust, and participative safety in teams are essential elements of teamwork but are not sufficient to result in fully effective teamwork. The authors emphasise the importance of extrinsic factors, such as health system policy and funding models, organization within practices, and the education of professionals [37].

Our study findings likewise strongly suggest that the formal framework plays a critical role in constituting a PHC team. However, PHC teamwork is not yet formalised in Lithuania, and it is often taken for granted. For example, the boundaries of GP and CN roles and responsibilities in team care remain fuzzy [38,39]. This situation could become a source of mutual dissatisfaction or even conflict among team members. For example, CNs seem to be floating between not being involved enough and being too involved in care due to the lack of clearly described duties for them. The findings here are consistent with the insights gained from a Canadian qualitative study that identified a lack of formal structures for supporting shared care practice and confusion about the roles and responsibilities of physicians, midwifes and nurses as being the essential barriers to inter-professional collaboration [40]. Studies by Halcomb et al. [41] and Pullon et al. [42] revealed a lack of consensus regarding the roles and scope of practice of PHC team members, which decreased PHC potential when responding to the growing demands of chronic care.

Our study indicates that the expectations GPs have regarding the actions of CNs in a team are very ambiguous. They would like to see CNs become more independent (i.e., gain more knowledge and responsibilities) while still adhering to fulfilling the tasks delegated by GPs. This duality on the part of GPs could be an indicator of a transition in PHC from being a health service delivery model dominated by GPs to a team-based goal-oriented model, where the patient’s views also gain more prominence. Nevertheless, it could also be a sign of the willingness of GPs to maintain the decision-making power within a PHC team. The fact that the topic of leadership was explicitly raised only in the CN discussions also could indicate the persisting dominance of GPs in PHC teams. Very likely that domination of GPs in the team is self-evident, the main shortcoming they see in this field – insufficient CNs obedience to fulfil tasks delegated by GPs. Other researchers also underline the defensiveness physicians feel to some degree about the changing roles of nursing professionals [43,44]. Furthermore, nurses distrust their own abilities to be more proactive in care [45]. Thus it is probable that a natural shift in the distribution of roles and responsibilities in PHC teams would take a long time, unless there is an explicit formal framework acting as an external influence [44].

A strengthening of the formal framework for teamwork should go hand-in-hand with interventions targeting the behavioural “software” of a team, because the “hardware” and “software” of teamwork seem to be interrelated. The individual behavioural aspects revealed in our study, such as communications among team members, respectful and non-hierarchical relationships, and team synergy could be strengthened via trainings, regular reflections and other team-building activities [46,47]. The existing research indicates the appropriateness of training for improving inter-professional attitudes, collaboration skills and collaborative behaviour [48]. Interdisciplinary collaboration trainings absolutely should target GPs in a role of collaborating with other disciplines, something that does not seem to be included often enough as a core task of physicians [49].

The data from this study suggest that the organisational environment could play an important role in increasing the effectiveness of teamwork. This insight is in line with the findings of other studies that a supportive team climate in an organisation increases team performance by increasing the members’ engagement in teamwork [20,50]. Future research should more consistently target the managerial issues for implementing inter-professional working models in health care in Lithuania, as was done in other countries [50,51]. The organisational aspects that the study participants revealed are important as a background for teamwork: optimising performance patterns for procedures, regulating patient flows, introducing innovations and having sensitivity to the individual needs of employees. All these aspects are, indeed, the general components of effective management. Although there is a high probability that effective PHC teamwork depends on effective management of the PHC institution itself, some especially sensitive aspects such as, for example, models for the financial compensations of team members should receive specific attention in future research. Our data are consistent with other study findings indicating that different funding models for different team members can become barriers to collaborative teamwork [40]. In general, funding models are closely related to teamwork efficacy [9,49]. Different approaches of financial compensations for different team members seem to become an “unfriendly” aspect in teamwork. Thus the findings of this study suggest that an organisation should be consistent in its principles for financially motivating all of the members in a team.

### Implications

This research has helped to identify elements that are related to teamwork in transforming primary health care in Lithuania. Our findings emphasise the necessity of establishing a formal framework – “hardware” for teamwork. Although the explicitness of formal background for team work, such as well-described functions and responsibilities of team members could be seen as limiting circumstance in highly integrative teams [18], in the initial stage of the implementation of team-based health care practice, when team collaboration is low, it seems to be an essential background for team functionality.

Teamwork “software” – the intrinsic or behavioural factors –plays a relatively more important role in more integrated teams. However, our findings suggest that it cannot be neglected in the initial developmental steps of team-based health care practice. In contrast, there seems to be a need for fragile teamwork “software” to be protected and fostered by the solid teamwork “hardware” in the beginning.

Although factors in the external or organizational environment apparently do not lose their importance in PHC systems with well-established teams [37], the team-friendly organizational environment is especially relevant in the initial stage of the development of team-oriented health care practices.

### Limitations

This study has its limitations – the views and experiences of the participants cannot be generalised to represent those in the greater primary health care community, since this study only included GPs and CNs working in the highly urbanised Kaunas region. GPs and CNs who practice in smaller towns or rural areas could have certain differences in their views; for example, the findings of Munro et al. [40] suggest that work in geographically isolated areas could exacerbate inter-professional tensions. Therefore future research should focus more on the experiences of rural GPs and CNs. An overwhelming majority of study participants were females; thus the male perspective on a PHC team could also be lacking. However, the gender structure of our study participants reflects the situation in Lithuanian PHC –more than 85% of GPs and almost 100% of CNs are females.

Another possible limitation of this study is that the focus group facilitators were GPs. Originally there was a decision to involve one moderator with a medical background and another with a nursing background, both trained in qualitative research methodology. However, just before the planned focus group discussions, the nurse investigator declined to participate in the project due to serious personal reasons and was replaced by a second GP. We took into account how the facilitators’ backgrounds could have affected the discussions with GPs and CNs differently. Thus, all transcripts were reviewed by the nurse investigator and all authors took part in the analytical process.

The third limitation concerns the decision to include only GPs and CNs as PHC teams members in the discussion. In our study the PHC team in fact is represented only by GP-CN dyads in their way from physician-cantered, hierarchical model to a change of roles and responsibilities between these two groups. According to study participants’ experience, PHC team structure seems to have more variety. However, our decision to limit study participants to GPs and CNs was made by drawing on formal descriptions of professionals constituting PHC team in Lithuania. Our findings indicating that the reality of the PHC team is wider that legislation could become an impulsion for wider reassessment of teamwork in PHC.

## Conclusions

There is broad consensus about the need for teamwork in PHC. However, the process of teambuilding is often taken for granted in Lithuanian PHC or not even considered as an issue. By providing insights that deepen the understanding of Lithuanian PHC teams, this study could induce specific policy changes to tackle weak points in teamwork.

This study reveals that when aiming to strengthen PHC teams, both formal and individual behavioural factors should be targeted. This study underscores the need in Lithuania to provide explicit formal descriptions of the roles and responsibilities of PHC team members and to determine the boundaries of their involvement. The training of team members is an essential component in the teambuilding process, although not sufficient by itself.

## Competing interests

The authors declare that they have no competing interests.

## Authors’ contributions

The work presented here was carried out in collaboration between all authors. LJ and IL contributed substantially to the conception and design of the study. LJ, IL and AK contributed substantially to the accumulation, analysis and interpretation of data. LJ drafted the manuscript. IL, AK, LV, GJ and LL were involved in drafting the manuscript, revising it critically and providing substantial content and rewriting support. All authors read and approved the final manuscript.

## Pre-publication history

The pre-publication history for this paper can be accessed here:

http://www.biomedcentral.com/1471-2296/14/118/prepub
